# GC–MS based targeted metabolic profiling identifies changes in the wheat metabolome following deoxynivalenol treatment

**DOI:** 10.1007/s11306-014-0731-1

**Published:** 2014-09-27

**Authors:** Benedikt Warth, Alexandra Parich, Christoph Bueschl, Denise Schoefbeck, Nora Katharina Nicole Neumann, Bernhard Kluger, Katharina Schuster, Rudolf Krska, Gerhard Adam, Marc Lemmens, Rainer Schuhmacher

**Affiliations:** 1 Department for Agrobiotechnology (IFA-Tulln), Center for Analytical Chemistry and Institute for Biotechnology in Plant Production, University of Natural Resources and Life Sciences, Vienna (BOKU), Konrad-Lorenz-Str. 20, 3430 Tulln, Austria; 2 Department of Applied Genetics and Cell Biology, University of Natural Resources and Life Sciences, Vienna (BOKU), Konrad-Lorenz-Str. 24, 3430 Tulln, Austria

**Keywords:** Metabolomics, Wheat (*Triticum aestivum*), Plant–pathogen interaction, Metabolism, Deoxynivalenol (vomitoxin), Fusarium head blight (scab), Phenylpropanoids

## Abstract

*Fusarium* *graminearum* and related species commonly infest grains causing the devastating plant disease Fusarium head blight (FHB) and the formation of trichothecene mycotoxins. The most relevant toxin is deoxynivalenol (DON), which acts as a virulence factor of the pathogen. FHB is difficult to control and resistance to this disease is a polygenic trait, mainly mediated by the quantitative trait loci (QTL) *Fhb1* and *Qfhs.ifa*-*5A*. In this study we established a targeted GC–MS based metabolomics workflow comprising a standardized experimental setup for growth, treatment and sampling of wheat ears and subsequent GC–MS analysis followed by data processing and evaluation of QC measures using tailored statistical and bioinformatics tools. This workflow was applied to wheat samples of six genotypes with varying levels of *Fusarium* resistance, treated with either DON or water, and harvested 0, 12, 24, 48 and 96 h after treatment. The results suggest that the primary carbohydrate metabolism and transport, the citric acid cycle and the primary nitrogen metabolism of wheat are clearly affected by DON treatment. Most importantly significantly elevated levels of amino acids and derived amines were observed. In particular, the concentrations of the three aromatic amino acids phenylalanine, tyrosine, and tryptophan increased. No clear QTL specific difference in the response could be observed except a generally faster increase in shikimate pathway intermediates in genotypes containing *Fhb1*. The overall workflow proved to be feasible and facilitated to obtain a more comprehensive picture on the effect of DON on the central metabolism of wheat.

**Electronic supplementary material:**

The online version of this article (doi:10.1007/s11306-014-0731-1) contains supplementary material, which is available to authorized users.

## Introduction

Fusarium head blight (FHB, scab) is a fungal plant disease commonly affecting wheat and other small-grain cereals. It is responsible for severe harvest losses and considered a relevant global hazard for food safety and security (Chakraborty and Newton 2011). The most important species provoking FHB is *Fusarium graminearum*, which is also known as the main producer of the trichothecene deoxynivalenol (DON). This mycotoxin constitutes a major virulence factor of the fungus and effectively inhibits eukaryotic protein translation (Pestka 2010). Besides the severe economic impact of FHB, due to direct yield losses, DON poses a health risk for consumers, and so also indirect costs for mycotoxin risk management are very high. The use of fungicides is generally regarded as inefficient to tackle FHB and trichothecene contamination alone (Lehoczki-Krsjak et al. 2010). Hence, breeding and planting of FHB resistant wheat lines is considered the most practicable and economical approach to manage the disease and its adverse impacts.

Resistance towards FHB in wheat is controlled by several quantitative trait loci (QTL) (Bai et al. 2003; Buerstmayr et al. 2009; Somers et al. 2003), with *Fhb1* (formerly *Qfhs.ndsu*-*3BS*) and *Qfhs.ifa*-*5A* being the most important ones (Anderson et al. 2001). Wheat cultivars containing the *Fhb1* QTL are already planted on a large scale in highly *Fusarium* affected areas in the United States (McMullen et al. 2012). *Qfhs.ifa*-*5A* contributes mainly to type I resistance, i.e. lowering the rate of initial infection. *Fhb1* and to a lesser extent also *Qfhs.ifa*-*5A* slows down or even inhibits the spread of the pathogen from the initial infection site (so-called type II resistance) (Schweiger et al. 2013). It has been proposed that the major resistance mechanism conferred by *Fhb1* is the conjugation of DON to DON-3-glucoside (Lemmens et al. 2005). This conjugation leads to an inactivation and much reduced toxicity of the conjugate towards plants is observed compared to the free toxin (Poppenberger et al. 2003). Recently, eight DON-biotransformation products were detected *in planta* besides the already known DON-3-glucoside using an untargeted screening strategy (Kluger et al. 2013). This includes a product annotated as DON-glutathione (GSH), further two DON-GSH-related metabolites (the processing products DON-S-cysteinyl-glycine and DON-S-cysteine) and five unknown DON conjugates. While *Fhb1* was associated with the formation of DON-3-glucoside, an association of GSH mediated detoxification with QTL is unknown to date. DON is a contributor to cellular injury and, besides inhibiting eukaryotic ribosomes, also damages plasma membranes and chloroplasts. Furthermore, it causes cell death in grains through the triggering of reactive oxygen species (ROS) such as hydrogen peroxide (Walter et al. 2010; Desmond et al. 2008). Walter et al. (2008) used wheat cDNA arrays (unfortunately covering only a small part of the genome) to analyse the effect of DON on the transcriptome of a cross between the cultivars ‘Remus’ and ‘CM-82036’. They discriminated ten transcripts related to proteins with various cellular functions and associated with the inheritance of DON resistance and *Fhb1*. In barley transcriptome analysis indicated a massive up regulation of cysteine biosynthesis upon DON treatment but also a strong up regulation of gene transcripts encoding ABC transporters, UDP-glucosyltransferases, cytochrome P450 s, and glutathione-*S*-transferases (Gardiner et al. 2010a).

Metabolomics has a great potential to substantially improve our understanding of plant–pathogen interactions, allowing the observation of plant mechanisms to resist pathogen induced stress and invasion based on small molecules (Kushalappa and Gunnaiah 2013). The application of GC-EI-MS in plant metabolite analysis has become routine during the past 10–15 years (Allwood et al. 2009) and is regarded as the gold standard for the analysis of primary metabolites (Fiehn et al. 2000; Hill and Roessner 2013). Modern metabolic profiling approaches offer the possibility to identify resistance-related metabolites (induced by pathogen challenge and differentially abundant and correlated with resistance), thereby contributing to an advanced understanding of their biosynthesis, regulation and the underlying genes (Fiehn et al. 2000). Despite the unquestionable importance of DON as virulence factor and thus FHB disease development, surprisingly to the best of our knowledge no studies have been conducted to investigate the specific role of DON on the wheat metabolome so far.

Therefore, in this study a suitable analytical platform was established and applied to investigate the plant’s response to the major fungal virulence factor DON at the metabolome level. We focused on the effect of DON rather than the overall response towards fungal infection on purpose, to be able to clearly attribute the observed metabolic changes to the toxin. Two parent wheat cultivars and four derived near isogenic lines (NIL), all previously studied with regard to their *F. graminearum* resistance level and characterized at the transcriptome level during infection (Kugler et al. 2013; Schweiger et al. 2013), were tested. The NILs were employed to investigate the influence of two major QTL associated with FHB resistance. A targeted approach was chosen to investigate whether metabolites are differentially abundant upon treatment and/or depending on the wheat genotype.

## Experimental

### Experimental design

#### Biosource

Six different spring wheat (*Triticum aestivum* L.) lines were used in the experiments. The resistant parent cultivar ‘CM-82036-1TP-10Y-OST-10Y-OM-OFC’ (abbreviated to ‘CM-82036’) originated from the cross ‘Sumai#3’/’Thornbird-S’ and was developed in a shuttle breeding program between CIMMYT Mexico and South America. It has a very high level of resistance against FHB comparable to ‘Sumai#3’ (Buerstmayr et al. 1996) and against DON (Lemmens et al. 2005). The second parent ‘Remus’ (‘Sappo’/‘Mex’//‘Famos’) is a spring wheat cultivar developed at the Bavarian State Institute for Agronomy in Freising, Germany. It possesses well-adapted agronomic characters for cultivation in Central Europe but is highly susceptible to *Fusarium* ear infection and DON (Buerstmayr et al. 1996; Lemmens et al. 2005). In addition four near isogenic wheat lines (NILs), which differed in two validated QTL (see Table 1) related to the FHB resistance level (*Fhb1* and *Qfhs.ifa*-*5A*) were included in the experiment (Schweiger et al. 2013). The NILs have been developed from one BC5F1 plant with ‘Remus’ as the recurrent parent (5 backcrosses). In the BC5F2 lines that contain the resistance alleles from ‘CM-82036’ of both *Fhb1* and *Qfhs.ifa*-*5A*, or either *Fhb1* or *Qfhs.ifa*-*5A* or none have been selected (Table 1).

**Table 1:** Distribution of the two QTL mainly responsible for FHB resistance in wheat in the two parent lines (‘Remus’, no QTL, susceptible; ‘CM-82036’, both QTL, resistant) and four near isogenic lines (NILs; C1–C4), all of which are near isogenic to the parent line ‘Remus’

Genotype		QTL *Fhb1*	QTL *Qfhs.ifa*-*5A*
Remus	Parent line	**−**	**−**
C1	NIL	**+**	**+**
C2	NIL	**+**	**−**
C3	NIL	**−**	**+**
C4	NIL	**−**	**−**
CM-82036	Parent line	**+**	**+**

#### Growth environment

Seeds of the spring wheat lines were germinated and the seedlings were submitted to a cold treatment at 5 degrees centigrade for one week to improve tillering. Pots (diameter 23 cm) were filled with 7 L of a homemade substrate (mix of 500 L heat-sterilized compost, 250 L peat, 10 kg sand and 250 g rock flour). In each pot 5 plants of the same wheat line were planted. During the experiment the pots were watered if required (typically 3 times/week). Water was applied until the substrate was completely wet and the water started to seep out through the holes in the bottom of the pot. The soil substrate contained sufficient minerals to support seedling growth. At the end of tillering (stage 5 on the Feekes scale) 2 g of a mineral fertilizer (COMPO Blaukorn^®^ ENTEC^®^, N/P/K/Mg: 14/7/17/2) was applied per pot. During plant cultivation the plants were treated twice a week overnight (10 h) with sulphur (S_x_) (sulphur evaporator, Nivola^®^) to prevent mildew. This treatment was stopped when heading started.

Experimental design was a completely randomized block with 5 (biological) replications. Upon reaching the correct developmental stage, the ears were labeled before treatment. Only one ear per plant was used in the experiment to prevent possible systemic effects of an earlier treated ear on the other later flowering ears of the same plant. Experiments were done in a greenhouse with computer-controlled settings for light, temperature and relative air humidity. Light was provided by a mix (50:50) of two types of lamps: 1) MASTER SON-T PIA Plus and 2) MASTER HPI-TPlus (Philips). Light intensity (outside dark) was 360 μmol s^−1^ m^−2^ at 1 m above the soil throughout the complete experiment. Relative air-humidity was set between 60 and 70 % during plant growth. Temperature (day/night) and duration of illumination (in hours) varied according to the development stage of the plants: after planting until the end of tillering (stage 3 on the Feekes scale), 12 °C/10 °C/12 h; end tillering until mid-stem extension when the ear starts to swell (stage 8), 14 °C/10 °C/14 h light; mid stem extension to start heading (stage 10.1), 16 °C/14 °C/14 h; from the start of heading until start of flowering (stage 10.51), 18 °C/14 °C/14 h; and from the start of flowering (stage 10.51) until the end of the experiment, 20 °C/18 °C/16 h. The seedlings needed 9–10 weeks to reach the flowering stage.

#### Treatment

As mentioned above the plants were illuminated for 16 h day^−1^ during treatment. The lamps were switched on at 05:00 am in the morning. Treatment of the ears started at 08:00 am. Flowering ears (stage 10.51) were selected and individually labeled. A woolen thread was used to label the lowest treated spikelet. In the left and the right floret of the treated spikelets 10 μL of the DON solution (5 g/L in water) was applied between the palea and the lemma directly on the floret using a multi-stepper pipette without wounding the plant. The central floret was not included. Ten adjacent spikelets (located in the center of the ear) starting with the labeled spikelet were treated in acropetal direction. In total, one milligram toxin was applied per ear, equally distributed in main 20 florets. Mock ears were treated exactly in the same way using bi-distilled water only. Subsequently the ear was covered for 24 h with a small transparent plastic bag internally wetted by spraying 2 mL of bi-distilled water with a hand-hold sprayer. This assured a high relative humidity promoting diffusion of the mycotoxin into the ear. Besides DON and mock treatment, wheat ears were also inoculated with a *F. graminearum* spore suspension as a third treatment in the course of this experiment. These results are beyond the scope of this paper and will be reported elsewhere.

#### Harvest

The treated ears were sampled either directly after treatment (time point 0) or 12, 24, 48 and 96 h after treatment. The ear was cut from the plant and the non-treated spikelets were carefully removed by clipping the rachis with a surgical scissor below and above the treated spikelets. The scissors were cleaned before collecting the next sample using 70 % ethanol. The treated ear part including the rachis and complete spikelets (chaff and florets) was put in a small vial. The vial was immediately filled with liquid nitrogen resulting in shock freezing of the sample. All treatments were carried out as five biological replicates except in four groups, where only four replicates were obtained. This resulted in 25 DON treated and 25 mock treated samples per genotype (50 samples per genotype, total number of biological samples: 296).

### Chemicals and reagents

Methanol (LiChrosolv, LC gradient grade) was obtained from Merck (Darmstadt, Germany) and acetonitrile (HiPerSolv Chromanorm, HPLC gradient grade) from VWR (Vienna, Austria). Water was purified by reverse osmosis using an ELGA Purelab Ultra-AN-MK2 system (Veolia Water, Vienna, Austria). DON (purity >98 %) was produced and purified according to Altpeter and Posselt (1994). Internal standards (^2^H_8_-valine, ^2^H_4_-succinic acid, ^13^C_6_-glucose, nonadecanoic acid methylester (NONA)), as well as formic acid (MS grade), chloroform, pyridine and methoxyamine hydrochloride (MOX) were purchased from Sigma Aldrich (Vienna, Austria), N-methyl-N-trimethylsilyl trifluoroacetamide (MSTFA) from Wagner Munz (Vienna, Austria). GC–MS reference standards were obtained either from Sigma Aldrich (Vienna, Austria) or as a gift from Dr. Nils Hoffmann (Bielefeld, Germany). Depending on the solubility, standard stock solutions were prepared in acetonitrile, 50 % aqueous methanol or water. Mixed n-alkane standard solutions C_8_–C_20_ and C_21_–C_40_ (Sigma Aldrich, Vienna, Austria) for RI calibration were diluted with isooctane to a final concentration of 5 mg/L per alkane.

### Sample preparation and GC–MS analysis

All sampling and sample preparation steps as well as the reporting of chemical analysis and metadata relative to data pre-processing, pre-treatment, processing, post-processing and interpretation were performed in full compliance with the metabolomics standards initiative (MSI) according to Fiehn et al. (2007), Goodacre et al. (2007) and Sumner et al. (2007). Harvested samples were immediately quenched in liquid nitrogen and subsequently stored at −80 °C until further processing. Each of the frozen wheat samples were milled to a fine powder for 2 min at 30 Hz using a ball mill (MM301 Retsch, Germany) with liquid nitrogen pre-cooled 10 mL stainless steel vessels and a 9-mm stainless steel ball. The homogenized wheat ears (100  ±  2 mg fresh weight) were weighed into Eppendorf tubes and extracted with 1 mL of pre-cooled methanol/water 3 + 1 (v/v) including 0.1 % formic acid, by vortexing for 10 s and further treatment in an ultrasonic bath at room temperature for 15 min according to De Vos et al. (2007). Samples were centrifuged for 10 min at 8,500×*g* at 4 °C and 200 μL of the supernatant were combined with 140 μL water, 75 µL chloroform and 10 µL of internal standard mixture (each 1.7 g/L ^2^H_8_-valine, ^2^H_4_-succinic acid, ^13^C_6_-glucose in methanol/water 1 + 1 (v/v)). After shaking, the tubes were again centrifuged for 4 min at 8,500×*g* at 4 °C and 200 µL of the methanol/water phase were transferred into micro-inserts in GC/HPLC vials. The samples were evaporated overnight using a centrifugal evaporator (Labconco, Kansas City, MO) at 15 °C and subsequently put on the GC auto sampler (PAL LHX-xt, CTC Analytics, Carrboro, NC) for an optimized online two step derivatization procedure. The sample was re-suspended in 40 µL MOX (20 mg/mL pyridine) and agitated for 90 min at 60 °C. Thereafter, 50 µL MSTFA and 10 µL internal standard (NONA, 370 mg/L) were added before the vial was again agitated at 60 °C for 60 min. Prior to analysis, the samples were kept in the drawer for 10 min at 4 °C to allow equilibration and sedimentation of particles. For separation and detection of analytes an Agilent 7890A gas chromatograph coupled to a 5975C inert XL MSD detector (Agilent, Waldbronn, Germany) was used. The instruments were controlled by ChemStation software (Agilent Technologies, version E.02.01.1177). A volume of 1 µL of the liquid sample was injected into the split/splitless injector at 250 °C in split mode and using a split of 25:1. An HP5-ms column (30 m × 0.25 mm × 0.25 µm; Agilent Technologies, Waldbronn, Germany) was operated at a constant flow of 1 mL/min helium. After keeping the oven at 50 °C for 2 min, it was heated to 310 °C (10 °C/min) and subsequently kept at this temperature for 15 min. The MSD interface was kept at 290 °C throughout the run. After 7 min of solvent delay, the detector was used in scan mode *m/z* 50–800. The EI source was kept at 230 °C and the MSD quadrupole at 150 °C. Autotuning of the MSD was performed at least once a week or after exchange of spare parts, such as the liner which was exchanged after a maximum of 70 injections. All samples were measured in a randomized manner.

### Quality control for GC–MS measurements

As a quality control measure, several QC sample types were employed, making up more than one third of all injections in total. First, homogenized and pooled wheat ears from the presented experiment, representing all genotypes, treatments and time points, were included for data normalization (referred to as *wheat aggregate QC sample*). Mixed n-alkane solutions for RI calibration were included regularly to ensure stable retention times (*RI calibration standard*). Furthermore, a QC standard mixture consisting of 15 authentic reference standards in pure solvent was injected regularly (*mixed standard QC sample*) after drying and derivatization. Besides, empty vials were either filled with the extraction solvent (*solvent blank QC*; dried before derivatization) or left empty (*empty vial QC*) and included into the measurement sequence to test for contaminations of the derivatization solvents or unintended analyte carry over from sample to sample, respectively.

### Data processing by *Metabolite Detector* software

Raw GC–MS data files were acquired by ChemStation and exported in the net.cdf format. The converted files were subsequently imported by the MetaboliteDetector software (Hiller et al. 2009; version 2.5.20130104-Linux) and further processed. Besides mass spectral deconvolution (settings: peak threshold 5; minimum peak height 5; deconvolution width 7; required number of peaks 20) and computation of feature areas for relative quantification, the retention index for compound recognition was calculated and the corresponding spectra were compared to an in-house library. This library was established by measuring 132 authentic reference standards using the same derivatization protocol, analytical parameters and instrument as the wheat samples analysed in the study at hand as recommended for unambiguous identification by Allwood et al. (2011). Metabolites were identified by comparison of mass spectra and retention indices to the spectra library using a cut-off value of 70 % (Abu Dawud et al. 2012). The feature areas of three specific fragment ions were recorded for each metabolite and the ion with the highest signal to noise ratio was detected by an in-house, Python based software, and chosen for further data processing and comparative quantification in all samples. In case of multiple silylation products per metabolite only the most abundant derivate was utilized for relative quantification after careful evaluation as suggested by Lisec et al. (2006). A table providing relevant metadata of chromatography and mass spectrometry as well as information on the identification level can be found in the Supplementary Material (Table S1).

### Data normalization

To compensate for MS intensity shifts between measurement sequences and genotypes, data normalization was performed before statistical analysis. To this end, for each experimental sample the integrated feature area of a single compound under investigation was divided by the average feature area of the respective metabolite in the wheat aggregate QC samples within a distinct measurement sequence. For metabolites which were not detected in the majority of wheat aggregate QC samples or for which the coefficient of variation (CV) of feature areas ranged above 30 % for the wheat aggregate QC samples, the average feature area of the ion at *m/z* 74 of the internal standard nonadecanoic acid methylester (NONA) in the wheat aggregate QC samples in a particular sequence was used for normalization. Moreover, all data were manually inspected during data evaluation to identify the underlying causes of missing values and outliers using MassHunter (Agilent Technologies, Qualitative Analysis B.06.00) and MetaboliteDetector software. The data matrix containing normalized metabolite abundances is available at the MetaboLights metabolomics data repository under study identifier MTBLS112 (http://www.ebi.ac.uk/metabolights/MTBLS112).

### Statistical analysis

The final data matrix was processed and evaluated by a tailored in-house R-scipt (R version 3.0.2 (R Development Core Team 2012) and R-Studio, Version 0.97.551). Missing values were replaced by the lowest value of the respective metabolite in the data set ±40 % random variation to keep the variance of the data set. Normal distribution was tested by the Shapiro–Wilk test followed by one Dean-Dixon outlier test per group after log transformation. Outliers were replaced by the median value ±20 % random variation of the respective group assuming that the metabolite abundances are similar within the same biological group (Hrydziuszko and Viant 2012).

The Šidák method (Šidák 1967) for multiple testing correction was applied before univariate comparison of individual metabolites between two treatments with a non-paired *t*-test. This correction prevents an accumulation of the global cumulative alpha-error by adapting the individual significance level of each performed t-test. The global significance threshold thus remained 5 %. The R-package ChemometricsWithR (Wehrens 2011) was used for principal component analysis (PCA). Metabolite feature areas were range-scaled across all experimental samples (Van den Berg et al. 2006) prior to PCA and ellipses in the PCA scores plot were calculated using the co-variance matrices of PC1 and PC2 of the respective sample types. For heatmap clustering of samples and metabolites the squared Euclidean distance and ward linkage were utilized.

## Results and discussion

### Overview of GC–MS analysis and workflow performance

#### Metabolite coverage

For the identification of metabolites an in-house library consisting of 132 compounds was established. We were able to detect 66 of those metabolites in the tested wheat samples on a regular basis (i.e. the metabolite was present in at least four out of five replicates for at least one time point). Authentic reference standards were purchased when feasible (i.e. available commercially and accessible by the chosen analytical approach). As most of the detected metabolites were annotated using a library created by reference standards, they can be regarded as “level 1 - identified compounds” when utilizing the four levels of metabolite identification proposed by the MSI (Sumner et al. 2007). We chose a targeted approach predominantly consisting of primary metabolites but also included metabolites that have putatively been related to FHB resistance before (Bollina et al. 2011; Hamzehzarghani et al. 2008). Some of the 66 annotated metabolites had to be excluded due to considerable carry over or metabolite levels close to the limit of detection. An example for this was e.g. the observation that a certain metabolite was detected in only one out of six measurement sequences, probably due to a better performance of the instrument on that particular day for the effected analyte. Therefore, the final data matrix contained a total number of 58 analytes (Table S1). When comparing the targeted with the untargeted processing option of the MetaboliteDetector software it was found that we were able to automatically annotate about 40 % of all features which were detected in case of the untargeted approach using the established in-house library. Taking into account that this workflow was tailor made to detect predominantly primary metabolites, we judge this as a share which is highly satisfying for a targeted approach.

GC–EI–MS instrumentation was employed to establish the applied analytical platform. For the required derivatization of polar metabolites in GC–MS based methods MSTFA and MOX are most often the reagents of choice and were also utilized in the workflow described here. An automated in-line derivatization and sample injection system was employed to guarantee timely derivatization at defined constant temperature just before analysis since this step was described as being very critical in the literature (Roessner and Dias 2013). However, artefact formation can occur during derivatization for GC–MS analysis. The loss of a guanidine group of agmatine during silylation with MSTFA results in the product putrescine-4TMS. In analogy, arginine also loses a guanidine group with ornithine-4TMS as product (Fig. S1). Thus, for the biogenic amines agmatine and putrescine the sum of both substances can be measured in the form of putrescine, while the sum of arginine and ornithine is measured in the form of ornithine by GC–MS. Also, citric and isocitric acid were evaluated as a sum parameter since both substances exhibit similar spectra and the column did not allow for a clear separation of the compounds under the applied conditions.

#### Results of QC measurements

Although the application of GC–MS in plant metabolite analysis has become routine (Allwood et al. 2009) every workflow needs adequate in-house performance evaluation and QC measures as this is crucial for the validity and repeatability of such studies. Adequate analytical accuracy was assured by use of aggregate wheat QC samples, which overall constituted more than 20 % of measured samples within a certain sequence and permitted to normalize all data. For 90 % of the abundant metabolites (i.e. detectable in at least 80 % of the samples) the feature areas, which were used for comparative quantification, showed a CV below 30 % in the aggregate wheat QC samples within all measurement sequences. For other GC–MS based plant profiling studies it was found that the variability due to biological variation is greater than the variability due to the overall analytical precision (Fiehn et al. 2000). RI calibration standards proofed stable retention behavior of the GC column within and across all measurement sequences and the solvent blank and empty vial QC samples verified the extraction and derivatization solvents not to be contaminated. Based on this evaluation we judged the technical workflow to be well suited for the performed experiment.

#### Data pre-processing (metabolite identification, missing value and outlier treatment)

The parameters of the employed MetaboliteDetector software (Hiller et al. 2009) were extensively tested and optimized using experimental wheat- and various different QC samples (wheat aggregate QC sample, mixed standard QC sample, QC solvent blank). For identification of metabolites a combined score of at least 70 % for mass spectra and RI index was utilized. The maximum RI difference for a feature in the library and the sample respectively was set to 10.

Missing values were replaced by the lowest value (Hrydziuszko and Viant 2012; Steuer et al. 2007) of the respective feature in the data set ±40 % random variation to keep the variance of the data. Less than 20 % of the feature areas in the matrix were affected by this procedure and thereof most missing values were observed for metabolites which were detected at later time points only. To further justify this approach, it was manually verified that the underlying chromatogram of this lowest value, assumed to be the limit of detection, corresponds to a signal to noise ratio of approximately 3 to 1.

The Dean-Dixon test revealed that less than 3 % of the data points in the matrix were regarded as outliers and affected by the median replacement procedure described above. Thereof, many were in groups with one missing value (out of five replicates) which were obviously not due to a missing feature in the raw data but because the identification criteria of the MetaboliteDetector were too rigorous (max RI difference 10). When the RI window was altered to 20, many of those missing features were additionally detected. However, when increasing the RI tolerance window the annotation might become more doubtful hence we favored this approach.

### General description of metabolic response in wheat

**Fig. 1:**
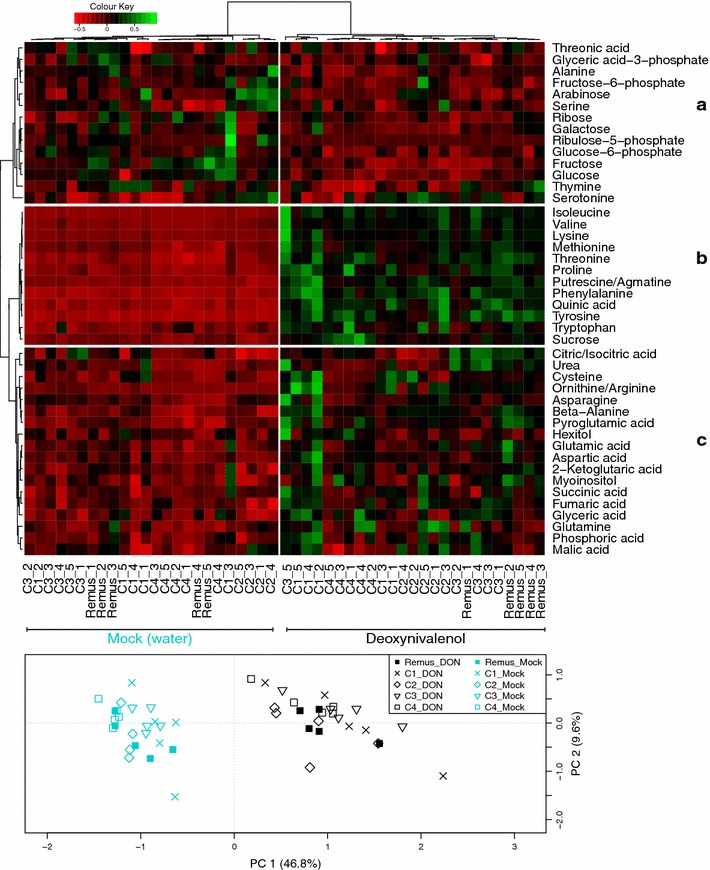
Heatmap and principal component analysis (PCA) illustrating the different metabolite levels in ‘Remus’ and its four NILs C1, C2, C3 and C4, 48 h after deoxynivalenol treatment. Metabolite feature areas were normalized and range-scaled across all experimental samples. Three different metabolite clusters can be identified in the heatmap: (**a**) contains metabolites, which tended to be less abundant as a result of the DON treatment when compared to mock, while cluster (**b**) shows an increase in metabolite levels. Cluster (**c**) contains metabolites which showed a moderate abundance increase following DON treatment. The clustering according to DON (*black*) and mock (*blue*) treatment was highly pronounced in the sample clustering and the PCA while genotypes clustered only occasionally. The first principal component (PC1) was inverted to comply with the heatmap (Color figure online)

**Fig. 2:**
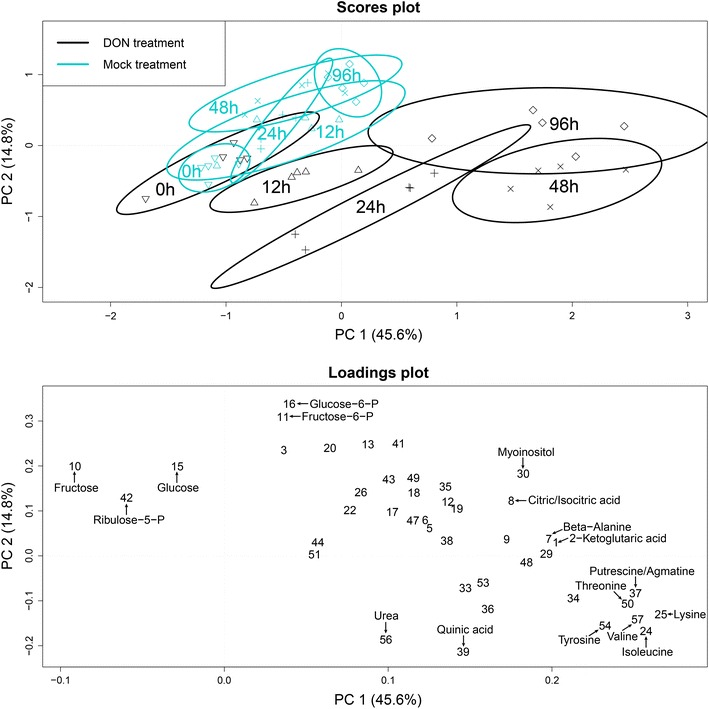
Scores and loadings plot of the performed principle component analysis (PCA) for the wheat line ‘Remus’ following deoxynivalenol and mock treatment. At time point zero the treatments overlap, indicating an identical state of the plants at the initial stage of the experiment. In contrast, the other sampling time points are clearly separated after deoxynivalenol treatment, thereby demonstrating its effect over time and proofing that an appropriate amount of toxin was applied to the plants. To enhance visibility only a selection of metabolites is enumerated in the loadings plot, the numbering of metabolites is in accordance with Table S1

As a first step to evaluate the final data matrix we run a set of principal component analysis (PCA) and compared DON and mock (i.e. water) treated samples at different time points in all six genotypes. As a result the ‘CM-82036’ cultivar was excluded in this initial evaluation as its different genetic background caused a clear separation from the other five genotypes and minimized the visibility of effects which potentially can be attributed to the QTL present in the different NILs. Using the PCA a clear distinction between mock and DON treated samples became obvious already at the first time point at 12 h after treatment. The variance increased until 24 h after treatment and likewise until 48 h after treatment at which the difference between mock and DON treated samples was most prominent (PCA in Fig. 1). 96 h after DON treatment the separation between the tested wheat lines, as based on the measured metabolites, was still very distinct but slightly decreased when compared to 48 h. Based on these observations we concluded that time point 48 h is the one most feasible to evaluate changes in metabolite patterns in detail. Furthermore, a PCA was performed to specifically evaluate the effect of the different time points in the cultivar ‘Remus’ after DON and mock treatment. At hour zero both treatments are overlapping thereby demonstrating that the plants were in an identical state at the beginning of the experiment. The distinct separation of the different sampling points following DON treatment further confirmed that the quantity of applied DON (1 mg per wheat ear) was appropriate as a continuous progress of the metabolic response over time was observed based on the levels of the measured metabolites (Fig. 2).

The dominating effect of the treatment over the genotype becomes evident in the heatmap (Fig. 1). The heatmap was generated for 44 metabolites which were found frequently across all genotypes. Three clusters were identified with distinct patterns of altered metabolite abundances.

The powerful effect of the fungal major virulence factor DON can also be seen in Fig. S2 where altered metabolite levels between mock- and DON treated samples of the wheat line ‘Remus’ are illustrated. Thirty-two of the detected metabolites were altered by the treatment when testing at a significance level of *p* < 0.05. The fact that 32 out of 66 metabolites (48 %) were significantly affected by the DON treatment highlights the ultimate impact of this single virulence factor and for the first time demonstrates its large influence on the metabolome level. When applying a multiple significance correction (Šidák 1967) this number decreased to eleven metabolites due to a more rigorous significance limit (*p* < 0.00091). Eight out of these eleven metabolites were amino acids. The others included the carbohydrate transport form sucrose, the biogenic amines putrescine/agmatine and urea. For further eight metabolites the *p* value could not be calculated since the analyte was absent in one of the treatments. For example shikimic acid was induced by DON whereas it was not detected in the mock treated ‘Remus’ samples (indicated in Fig. S2 as “not detected” (n.d.)). The NIL C4, exhibiting the highest genetic similarity to ‘Remus’, showed exactly the same behavior for highly significant metabolite alterations with the exception that additionally aspartic acid was more abundant in DON treated samples (*p*-value: 0.0008).

### Effect of deoxynivalenol on central metabolism

**Fig. 3:**
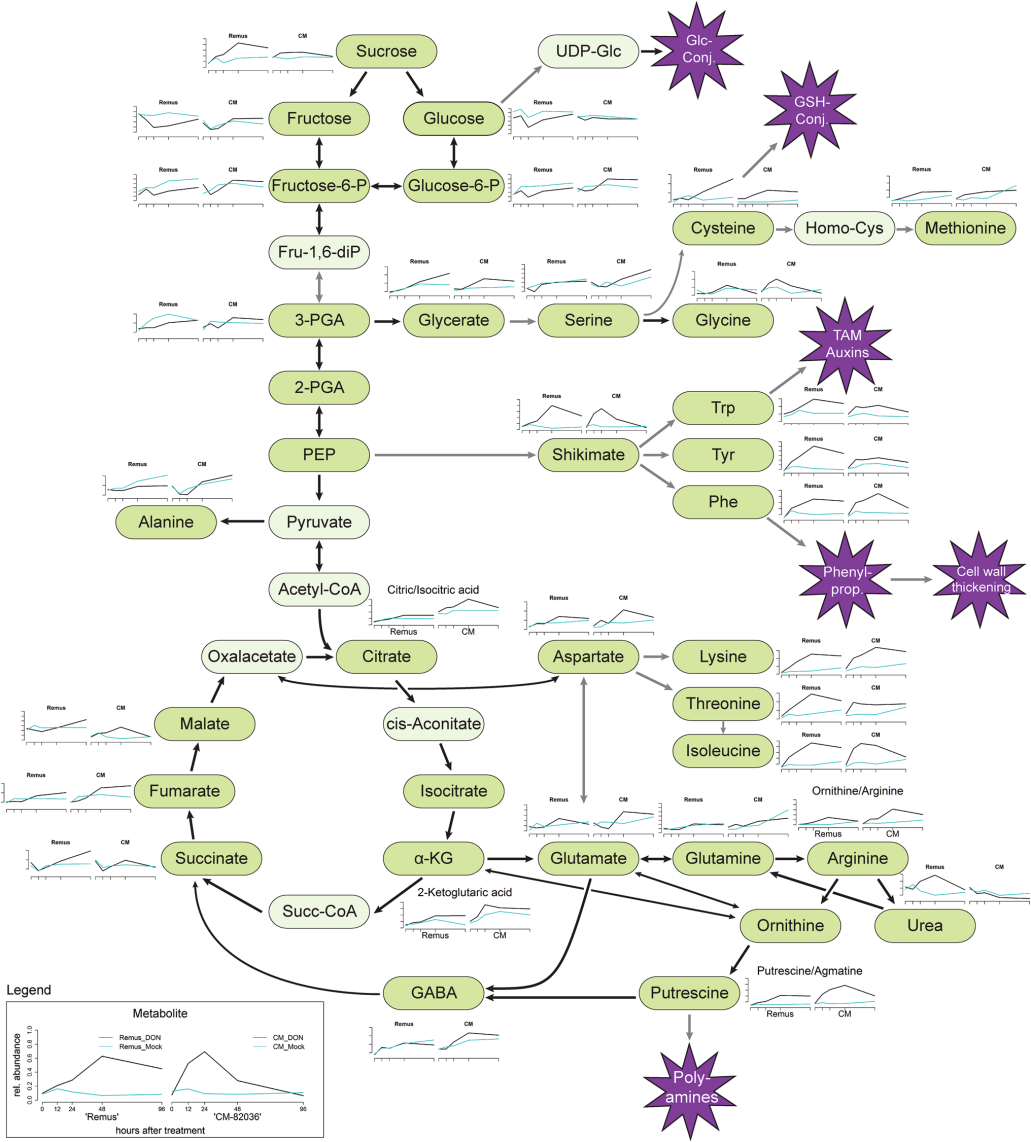
Schematic overview of metabolic pathways upon deoxynivalenol (*black*) and mock (*blue*) treatment in the wheat cultivars ‘Remus’ and ‘CM-82036‘. *Black arrows* indicate specific metabolic steps whereas *grey arrows* stand for multi-step reactions. Metabolites in faint colour were not included in the measurements. Abbreviations: *3-PGA* 3-phosphoglycerate, *2-PGA* 2-phosphoglycerate, *α-KG* α-ketoglutarate, *Fru-1,6-diP* fructose-1,6-diphosphate, *GABA* 4-aminobutyrate (γ-aminobutyrate), *Glc-Conj.* Glucoside-conjugates, *GSH-Conj* Glutathione-conjugates, *Homo-Cys* Homocysteine, *PEP* phosphoenolpyruvate, *Phe* phenylalanine, *Succ-CoA* succinyl-CoA, *TAM* Tryptamine, *Trp* Tryptophan, *Tyr* Tyrosine, *UDP-Glc* uridine diphosphate-glucose (Color figure online)

A number of metabolites which showed tendencies towards lower concentrations following DON treatment in ‘Remus’ and its NILs (Fig. 1, cluster A) participate in glycolysis (glucose, glucose-6-phosphat, fructose-6-phosphate, glyceric acid-3-phosphat). Also other sugars (fructose, galactose, ribose, arabinose) and a sugar phosphate (ribulose-5-phosphat) followed this trend. Ribulose-5-phosphate and fructose are also mainly responsible for the separation of the initial time point in the PCA depicted in Fig. 2 (see loadings plot). Only two amino acids, namely alanine and serine, were less abundant in the DON treated samples. Interestingly, both are known to have a high turnover and are closely connected to glycolysis: serine can be produced from glyceric acid-3-phosphate and alanine is most commonly generated by a reductive amination of pyruvate (Fig. 3).

In contrast, several amino acids showed a clearly elevated level as a response to DON treatment (Fig. 1, cluster B). Nine of the twelve metabolites in this cluster were amino acids. Furthermore, increased levels of sucrose were observed, which presumably indicates carbohydrate transport from photosynthetic tissue to the sink tissue in the ear. Besides, also quinic acid (a precursor of the shikimate pathway) and putrescine/agmatine occurred at higher abundances in the DON treated group and were therefore attributed to cluster B. These results indicate that the toxin alone is able to elicit at least parts of the pathogen defense program observed during *Fusarium* infection.

**Fig. 4:**
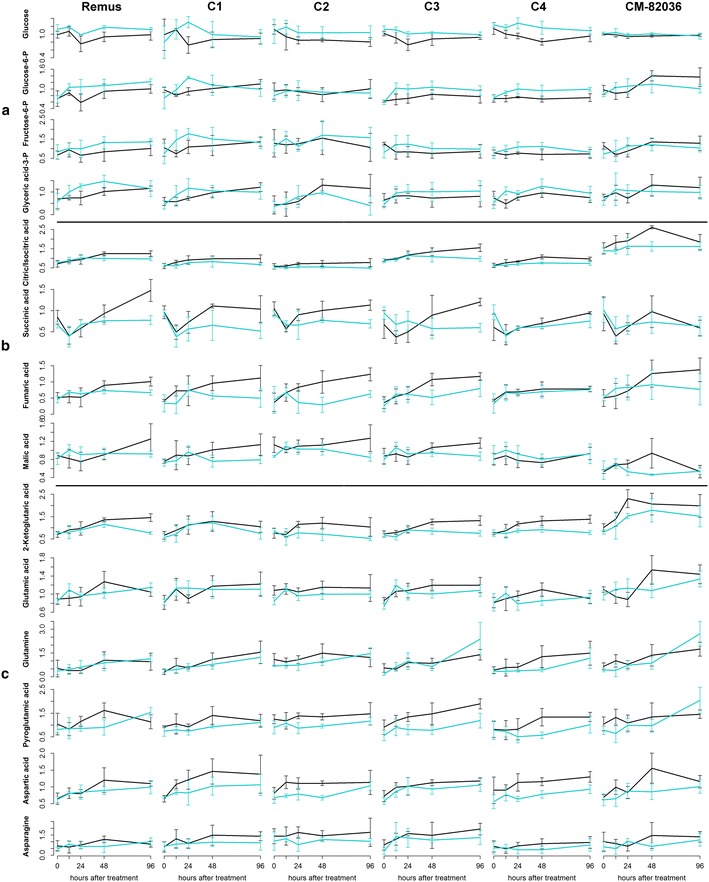
Time line of metabolite abundances of glycolysis intermediates (**a**), TCA cycle metabolites (**b**) and derived keto acids, amino acids and amines (**c**). Deoxynivalenol treatment is depicted in *black*, whereas mock treated samples are *blue*; whiskers describe the standard deviation. The* y-axis* gives the normalized relative metabolite abundance. Glycolysis products exhibited decreased levels after DON treatment (**a**) while molecules included in the TCA cycle (**b**) and derived keto acids, amino acids and amines (**c**) seemed to be stimulated by treatment (Color figure online)

The heatmap cluster (C) contains metabolites which exhibited moderate concentration increases following DON treatment. Interestingly, a number of metabolites in this cluster belong to the TCA cycle (citric/isocitric acid, succinic acid, fumaric acid, malic acid). In Fig. 4 the increase in TCA intermediates and the decrease of glycolysis related metabolites is illustrated in detail for all investigated genotypes. These observations could point at the need of the plant to generate energy and reduction equivalents to cope with the toxin. Also after the application of the SnToxA effector protein onto wheat (another fungal toxin produced by the pathogen *Stagonospora nodorum*) photosynthesis decreased and metabolites involved in the TCA cycle increased in general (Vincent et al. 2011). The activation of plant defence actions requires an enhanced energy supply that presumably is mainly derived from photosynthesis, but also seems to require greater respiratory rates (Bolton 2009). In line with the moderate increase in TCA cycle intermediates, also derived keto acids which are precursors of amino acids were elevated. For example 2-ketoglutaric acid is used to form glutamic acid through transamination and both metabolites are increased upon DON treatment under the tested conditions. In analogy, glutamine and pyroglutamic acid (a derivative where the free amino group of glutamine or glutamic acid is cyclized to a lactam) as well as aspartic acid and asparagine were more abundant.

In plants ornithine and urea are typically derived from arginine via arginase in the mitochondrion. This arginine catabolism is crucial for the mobilization of nitrogen from source tissue (Witte 2011). The generated urea is exported to the cytosol, hydrolyzed, and the resulting ammonium is re-assimilated by glutamine synthase (Witte 2011). The loadings in Fig. 2 indicate that the abundances of ornithine and urea considerably changed over time in the ‘Remus’ genotype. It can be concluded that both the primary carbohydrate metabolism and the primary nitrogen metabolism of the plant were significantly modified by DON treatment (Fig. 3). However, the clear trend of decreased glycolysis intermediates and increased sucrose abundances following treatment was only evident in ‘Remus’ and all of the tested NILs. Therefore, this behavior appeared to be independent from the presence of the two resistance QTL. In the highly resistant parent line ‘CM-82036’ the observed effects were less pronounced. This suggests that additional factors, which are not associated with either of the two QTL help to protect ‘CM-82036’ against the toxic effects of DON.

### Effect of deoxynivalenol on metabolites specifically related to plant stress and plant defense

Some of the amino acids which were explicitly more abundant in DON treated wheat (Fig. 1, Cluster B) have been associated with different plant defense mechanisms. This includes the three aromatic amino acids phenylalanine, tyrosine, and tryptophan which are all synthesized by the shikimate pathway. This pathway is known to provide the building blocks for important aromatic secondary metabolites such as phenylpropanoids or other defense related compounds such as amines (e.g. tryptamine, tyramine) and its hydroxycinnamic acid amide conjugates as well as the plant hormone auxin (Fig. 3). Phenylalanine was described as FHB resistance related metabolite before in a study utilizing LC-HRMS (Gunnaiah et al. 2012). Methionine is a precursor of ethylene formation via the Yang cycle (Van de Poel et al. 2012) and was suggested as an inducer of plant defense responses. Interestingly, a recent experiment revealed three sulphur containing DON conjugates *in planta*: the glutathione conjugate (DON-GSH) and its processing products DON-*S*-cysteinyl-glycine and DON-*S*-cysteine (Kluger et al. 2013). The common increase of methionine and cysteine following DON application points to an important role of sulphur containing amino acids in glutathione-mediated DON detoxification. The intracellular cysteine pool was seemingly not depleted through the formation of DON-GSH, although GSH derived DON conjugates have been found in the same set of DON treated samples using LC-HRMS instrumentation (Kluger et al. unpublished).

**Fig. 5:**
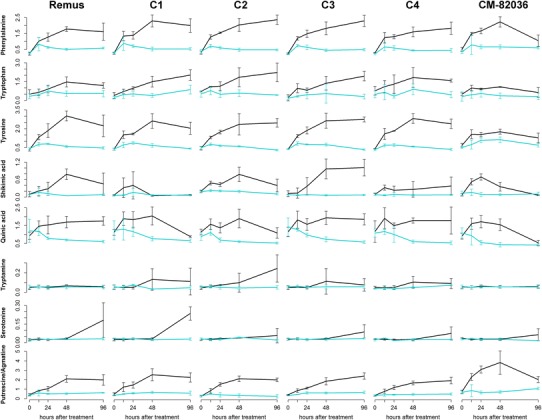
Time course of metabolites which generally have been associated with resistance towards Fusarium head blight in the two parent wheat lines and the four near isogenic lines C1–C4. Deoxynivalenol treatment is depicted in *black*, whereas mock treated samples are *blue*; whiskers describe the standard deviation. Most of the displayed metabolites were considerably more abundant in the DON treated samples. The highly resistant cultivar ‘CM-82036’ seemed to be faster in the induction of some of the metabolites, e.g. shikimic acid or putrescine/agmatine are enhanced already 12 h after treatment (Color figure online)

Furthermore, lysine, threonine as well as proline, which is known to protect plants against abiotic stress, were clearly more abundant in DON treated wheat ears (Fig. 1 and Fig. S3). Two biogenic amines, putrescine/agmatine, which originate from the amino acids ornithine and arginine, respectively, occurred at higher abundances in the DON treated group as well. Putrescine, which was tentatively identified in a previous study, was associated with increased *Fusarium* resistance (Hamzehzarghani et al. 2008). Its time course is shown for all tested wheat lines in Fig. 5 together with other metabolites which are generally presumed to be related to resistance.

Pyroglutamic acid, aspartic acid, and arginine have been reported to be associated with *Fusarium* resistance in an LC-HRMS approach before (Bollina et al. 2010) and were also found to be moderately elevated following DON treatment in this experiment. While in the mentioned study these metabolites have only been annotated putatively we can confirm these former findings by similar observations of a relative increase of their concentration levels and definitive identification (level 1). Pyroglutamic acid (also known as 5 oxo-proline) plays an important role maintaining GSH levels by glutamate recycling (Paulose et al. 2013).

### Metabolites related to the resistance QTL *Fhb1* and *Qfhs.ifa*-*5A*

One of the objectives of this experiment was to investigate if primary metabolites covered by the utilized method can be associated with the two major QTL responsible for FHB resistance in wheat. Therefore, we examined by univariate comparison the fold change of metabolite abundances between ‘Remus’ and the respective NILs 48 h after treatment. However, under the tested conditions no significant effect of one of the QTL on primary metabolite abundances was observed after 48 h. This can be explained by the dominating effect of the treatment rather than that of the tested QTL as derived from multivariate statistics (PCA and hierarchical clustering in Fig. 1). The lack of a QTL specific effect is in line with transcriptomics data of *Fusarium* infection of the same NILs (Schweiger et al. 2013), where also no clear evidence for a QTL specific effect on metabolic genes was found. Modified metabolic patterns potentially mediated by the QTL are probably more pronounced when looking at secondary metabolites particularly involved in pathogen defense. Hence, we intend to investigate those metabolites using different analytical methodology in the future (Bueschl et al. 2014).

Another potential explanation for the absence of significantly altered metabolite abundances which can be attributed to the tested QTL after 48 h could be the chosen time point. Since the highly resistant cultivar ‘CM-82036’ seems to have a generally faster metabolic response to fungal infection (Fig. 5), the time courses for both parent lines and the four NILs are plotted for all 58 annotated metabolites (Fig. S3) to allow also interpretation of earlier time points. Interestingly, shikimic and quinic acid seem to increase faster in genotypes containing *Fhb1* but then decrease after 48 and 96 h while in the non *Fhb1* containing genotypes these two metabolites reach a plateau at a constant elevated level throughout most of the observed time points. The decrease in metabolite levels at later time points could either indicate that the plants defense resources are becoming depleted or that defense via these metabolites is not active any more. However, this finding needs further investigation. Besides this observation, no clear QTL specific changes were obvious. It was already suggested by other studies that the pace of the plants defense response is more critical in determining resistance towards DON and thus the spread of FHB disease than the involved compounds itself (Walter and Doohan 2011). This was also observed for the wheat lines tested in this experiment on the transcriptome level (Schweiger, personal communication).

GC–MS mostly detects primary metabolites which frequently constitute intermediates of potential secondary metabolites. Theoretically, the pool of a biosynthetic precursor of a secondary metabolite could be stable, although a higher net flux to the relevant secondary metabolite occurs, if it is compensated by a higher flux from earlier biosynthetic steps. So we cannot exclude that different accumulation of defense metabolites is causing the difference in resistance due to the QTL, but this seems rather unlikely.

### Putative biological functions of the metabolic response induced by DON

A reduced amount of assimilates was observed following DON treatment. It has been previously described that DON induces (by unknown mechanisms) a light dependent loss of chlorophyll (Bushnell et al. 2009). Bleaching after DON application due to a reduction in chlorophyll content was also observed in duckweed (Abbas et al. 2013). In many plant-pathogen systems, photosynthesis decreases at the site of infection (Bolton 2009). We propose that the observed reduction in the amount of assimilates is caused by a reduction of photosynthesis. It might be possible that acetyl-CoA is produced via b-oxidation of fatty acids in the glyoxysomes once photosynthesis is impaired by DON. Acetyl-CoA could then be further utilized in the glyoxylate cycle, a pathway similar to the TCA cycle, to supply energy. Mature plants, including wheat, have been demonstrated to possess the enzymes involved in this cycle (Vincent et al. 2011). Therefore it is intended to evaluate the levels of fatty acids following DON application in further studies. The local reduction in photosynthetic metabolism initiates the transition from source status to sink status in infected leaf tissue (Bolton 2009). Interestingly, although the flowering wheat ear already constitutes a sink organ, we observed elevated sucrose concentration in the DON treated tissue. It should be mentioned that some pathogens can also drain energy from the primary metabolism to its own advantage (Bolton 2009; Seifi et al. 2013). For instance, hijacking sugar efflux with TAL effectors for increased loading of sucrose into phloem transport is a strategy of *Xanthomonas* on rice (Chen 2014). Hence, this DON induced mechanism could ensure a useful supply of the carbohydrate via long-distance transport. However, with respect to the progression of the FHB disease it might be more relevant that the observed increased sucrose abundance following DON treatment reflects the need of the plant to replenish UDP-glucose via the reverse reaction of sucrose synthase. UDP-glucose is utilized (and would otherwise be depleted) by highly DON induced UDP-glucosyltransferases which synthesize the conjugate DON-3-glucoside. It has been proposed that the major resistance mechanism conferred by *Fhb1* is the conjugation of DON to DON-3-glucoside (Lemmens et al. 2005). However, Gunnaiah et al. (2012) indicated an alternative mode of *Fhb1* mediated resistance related to cell wall thickening caused by the deposition of hydroxycinnamic acid amides, phenolic glucosides and flavonoids. In our study both DON-3-glucoside (Kluger et al. unpublished) and the abundances of shikimate pathway intermediates (Fig. 3) increased faster upon DON treatment indicating that both mechanisms actively contribute to resistance, and correlate with the presence of the *Fhb1* QTL. To further investigate these hypotheses we intend to perform further studies using isotopically labelled tracers which for example can be applied onto source tissue. Such a setup would allow to elucidate whether the tracer is translocated to the infection site. Furthermore, it is planned to determine the concentrations of UDP-glucose in the same set of samples using a targeted LC–MS/MS approach and external calibration for absolute quantification.

It seems plausible that the (partial) inhibition of protein synthesis by DON is of advantage to the fungus since the plant cannot efficiently translate induced defense transcripts into functional proteins. Moreover, blocking of protein synthesis by DON results in the accumulation of truncated proteins, which have to be turned over. It has been shown that ubiquitin-mediated degradation is important for DON resistance in yeast (Abolmaali et al. 2008). Theoretically, a block of protein biosynthesis and increased hydrolysis of aberrant proteins could lead to accumulation of free amino acids. In this scenario the proportion of the individual amino acids contained in the proteins should be unchanged by the turnover. Yet, we observed that only selected amino acids, not all, were increased. We therefore consider it more likely that the observed changes on the amino acid pools reflect changes in the transcription of biosynthetic genes, similar to responses which were also observed during *Fusarium* infection (Schweiger et al. 2013; Gardiner et al. 2010a). Unfortunately, no transcriptome derived data of DON treated wheat is available to verify this assumption.

Since the basal expression of most amino acid biosynthetic genes is already high, the frequently applied threshold of a two-fold change is often not reached in transcriptomic studies and as a consequence this increase is generally neglected. A reported exception is the extremely high up-regulation of cysteine biosynthesis in DON treated barley (Gardiner et al. 2010a). The active role of cysteine also in wheat is suggested by the fact that this amino acid was found in our study at constantly elevated levels throughout the experiment while DON-GSH and derived conjugates were continuously increasing, especially in the genotypes not carrying the *Fhb1* QTL (‘Remus’, C3, C4; data not shown). This indicates that the intracellular cysteine pool was not depleted but could be replenished in our plants. The other accumulating amino acids, in particular the aromatic amino acids may be further metabolized to synthesize defense compounds. The tryptophan biosynthetic gene anthranylate synthase was found transcriptionally up-regulated 5.9 × in DON treated barley after 24 h (Gardiner et al. 2010a). We also observed the simultaneous increase of phenylalanine, quinic acid and shikimic acid upon toxin treatment, which are all precursors of phenylpropanoids.

Furthermore, our results show that DON induces a higher level of various biogenic amines. It was shown that after infection with *F. graminearum* the glutamine-oxoglutarate aminotransferase cycle was redirected toward the production of ornithine and arginine, resulting in the formation of polyamines (Gardiner et al. 2010b). The induction of the conversion of agmatine to polyamines, which can induce DON production in *Fusarium* (Gardiner et al. 2009), was previously reported by Gunnaiah et al. (2012). Polyamines have many ascribed functions in abiotic and also biotic stress, in particular also in the formation of cell wall reinforcements by formation of hydoxycinnamic adic amides (Bassard et al. 2010). Such conjugates were indeed detected in an accompanying study using LC-HRMS (Kluger et al. unpublished).

## Concluding remarks

This paper describes the altered metabolic signature of six wheat genotypes carrying different combinations of the two most important FHB resistance QTL following DON treatment. The newly established GC–MS based metabolomics workflow including greenhouse experiments, sample preparation and analysis as well as the data processing and interpretation allows investigating the central wheat metabolism and will be a valuable tool for future investigations of other plant systems as well.

Many metabolites related to DON treatment were detected, but no clear effect of the QTL was found besides the faster increase in shikimate pathway intermediates in *Fhb1* genotypes. Our results indicate that photosynthesis is impaired by DON, and that both the primary carbohydrate metabolism and the primary nitrogen metabolism of the plant are significantly modified by DON treatment. Significantly increased amino acid levels were detected and may arise from hydrolysis of irregular proteins but are most likely due to an active defense response of the plant triggered by DON alone. Based on the obtained results we hypothesize that both, detoxification of DON and DON induced cell wall reinforcement are involved in the *Fhb1* mediated response towards DON treatment. Further studies are necessary to investigate if this also holds true for *Fusarium* infected wheat.

## Electronic supplementary material

Below is the link to the electronic supplementary material.

Supplementary material 1 (DOCX 24 kb)

Supplementary material 2 (TIFF 1396 kb)

Supplementary material 3 (TIFF 269 kb)

Supplementary material 4 (TIFF 316 kb)
