# Supplementary material for: GC–MS based targeted metabolic profiling identifies changes in the wheat metabolome following deoxynivalenol treatment
Source: Metabolomics. 2014 Sep 27;11(3):722–38. doi: 10.1007/s11306-014-0731-1 (PMC4419159; doi:10.1007/s11306-014-0731-1)
Supplement: Supplementary file 1 — Supplementary material 1 (DOCX 24 kb) [file 11306_2014_731_MOESM1_ESM.docx]

**Supplementary Material**

**Table S1.** Parameters of the 58 metabolites identified in the investigated wheat samples (n ≥ 4 out of five replicates in at least one experimental condition or time point). The peak area of the quantification ion was used for comparative quantification.

|  | **Metabolite** | **Derivate** | **PubChem CID** | **Retention Time** | **RI Index** | **Quantification Ion [*m/z*]** | **Qualification Ions [*m/z*]** | **Identification Level^a^** |
| --- | --- | --- | --- | --- | --- | --- | --- | --- |
| 1 | 2-Ketoglutaric acid | 1 MOX 2 TMS | 9601828 | 15.44 | 1589 | 198 | 288, 304 | 1 |
| 2 | Adenosine | 4 TMS | 524923 | 25.38 | 2672 | 230 | 236, 245 | 1 |
| 3 | Alanine | 2 TMS | 520350 | 8.95 | 1110 | 116 | 190, 117 | 1 |
| 4 | Arabinose | 1 MOX 4 TMS | 1453812-35-5 (CAS) | 16.57 | 1688 | 103 | 277, 307 | 1 |
| 5 | Asparagine | 3 TMS | 552067 | 16.56 | 1687 | 116 | 132, 231 | 1 |
| 6 | Aspartic acid | 2 TMS | 529617 | 13.52 | 1432 | 160 | 130, 174 | 1 |
| 7 | Beta-Alanine | 3 TMS | 521597 | 13.59 | 1438 | 174 | 248, 290 | 1 |
| 8 | Citric/Isocitric acid | 4 TMS | 553132/528659 | 18.24 | 1845 | 273 | 363, 375 | 1 |
| 9 | Cysteine | 3 TMS | 530165 | 15.22 | 1570 | 220 | 218, 221 | 1 |
| 10 | Fructose | 1 MOX 5 TMS | 20845408 | 18.89 | 1908 | 103 | 217, 307 | 1 |
| 11 | Fructose-6-phosphate | 1 MOX 6 TMS | 5378839 | 22.98 | 2362 | 315 | 299, 217 | 1 |
| 12 | Fumaric acid | 2 TMS | 5353016 | 12.47 | 1352 | 245 | 143, 246 | 1 |
| 13 | Galactose | 1 MOX 5 TMS | 5378916 | 19.1 | 1929 | 319 | 217, 205 | 1 |
| 14 | Gamma-aminobutyric acid | 3 TMS | 529208 | 14.88 | 1542 | 174 | 304, 175 | 1 |
| 15 | Glucose | 1 MOX 5 TMS | 20836750 | 19.17 | 1937 | 319 | 103, 205 | 1 |
| 16 | Glucose-6-phosphate | 6 TMS | 10357921 | 23.09 | 2375 | 387 | 315, 299 | 1 |
| 17 | Glutamic acid | 3 TMS | 519202 | 15.96 | 1634 | 246 | 128, 348 | 1 |
| 18 | Glutamine | 3 TMS | 21632770 | 17.67 | 1789 | 156 | 245, 155 | 1 |
| 19 | Glyceric acid | 3 TMS | 520886 | 12.36 | 1344 | 189 | 133, 292 | 1 |
| 20 | Glyceric acid-3-phosphate | 4 TMS | 553112 | 18.13 | 1834 | 357 | 299, 227 | 1 |
| 21 | Glycine | 2 TMS | 530141 | 9.33 | 1140 | 102 | 176, 204 | 1 |
| 22 | Hexitol | 6 TMS | 518921 | 19.48 | 1968 | 205 | 217, 319 | 2 |
| 23 | Homoserine | 3 TMS | 612634 | 13.9 | 1463 | 218 | 128, 219 | 1 |
| 24 | Isoleucine | 2 TMS | 21632765 | 11.53 | 1284 | 158 | 218, 159 | 1 |
| 25 | Lysine | 4 TMS | 553497 | 19.22 | 1942 | 174 | 317, 434 | 1 |
| 26 | Malic acid | 3 TMS | 522155 | 14.41 | 1503 | 233 | 245, 335 | 1 |
| 27 | Malonic acid | 2 TMS | 87656 | 10.5 | 1211 | 147 | 233, 148 | 2^b^ |
| 28 | Maltose | 1 MOX 8 TMS | 519729 | 26.41 | 2818 | 361 | 204, 217 | 1 |
| 29 | Methionine | 2 TMS | 520351 | 14.76 | 1532 | 176 | 293, 128 | 1 |
| 30 | Myoinositol | 6 TMS | 520232 | 21 | 2131 | 305 | 217, 318 | 1 |
| 31 | Myoinositol-phosphate | 7 TMS | 553143 | 23.87 | 2472 | 318 | 315, 387 | 1 |
| 32 | Norleucine | 2 TMS | 553200 | 11.81 | 1304 | 158 | 218, 159 | 1 |
| 33 | Ornithine/Arginine | 4 TMS | 588340 | 18.17 | 1838 | 142 | 174, 420 | 1 |
| 34 | Phenylalanine | 2 TMS | 522502 | 16.05 | 1642 | 218 | 192, 219 | 1 |
| 35 | Phosphoric acid | 3 TMS | 25317 | 11.55 | 1285 | 299 | 314, 300 | 1 |
| 36 | Proline | 2 TMS | 522501 | 11.85 | 1306 | 142 | 216, 143 | 1 |
| 37 | Putrescine/Agmatine | 4 TMS | 13483149 | 17.29 | 1755 | 174 | 214, 175 | 1 |
| 38 | Pyroglutamic acid | 2 TMS | 11807870 | 14.8 | 1535 | 156 | 258, 230 | 1 |
| 39 | Quinic acid | 5 TMS | 6427786 | 18.74 | 1894 | 345 | 255, 346 | 1 |
| 40 | Ribitol | 5 TMS | 14534972 | 17.19 | 1745 | 217 | 319, 205 | 1 |
| 41 | Ribose | 1 MOX 4 TMS | 9601869 | 16.72 | 1701 | 103 | 307, 217 | 1 |
| 42 | Ribulose-5-phosphate | 1 MOX 5 TMS | 553819 | 21.29 | 2163 | 357 | 299, 358 | 1 |
| 43 | Serine | 3 TMS | 522136 | 12.75 | 1373 | 204 | 218, 205 | 1 |
| 44 | Serotonine | 4 TMS | 599836 | 24.01 | 2491 | 174 | 290, 175 | 1 |
| 45 | Shikimic acid | 4 TMS | 609197 | 18.07 | 1829 | 204 | 255, 205 | 1 |
| 46 | Spermidine | 5 TMS | 55429-74-8 (CAS) | 22.32 | 2282 | 144 | 174, 116 | 1 |
| 47 | Succinic acid | 2 TMS | 520988 | 12.03 | 1320 | 247 | 262, 172 | 1 |
| 48 | Sucrose | 8 TMS | 632328 | 25.64 | 2707 | 361 | 217, 362 | 1 |
| 49 | Threonic acid | 4 TMS | 528672 | 15.36 | 1582 | 292 | 220, 205 | 2^b^ |
| 50 | Threonine | 3 TMS | 529620 | 13.12 | 1401 | 218 | 117, 219 | 1 |
| 51 | Thymine | 2 TMS | 81705 | 13.27 | 1413 | 255 | 270, 120 | 1 |
| 52 | Tryptamine | 3 TMS | 599834 | 22.04 | 2250 | 174 | 361, 175 | 1 |
| 53 | Tryptophan | 3 TMS | 21632772 | 22.02 | 2247 | 202 | 291, 203 | 1 |
| 54 | Tyrosine | 3 TMS | 529998 | 19.4 | 1960 | 218 | 280, 179 | 1 |
| 55 | Uracil | 2 TMS | 82640 | 12.43 | 1349 | 241 | 255, 256 | 1 |
| 56 | Urea | 2 TMS | 87562 | 10.97 | 1244 | 189 | 171, 99 | 2^b^ |
| 57 | Valine | 2 TMS | 11108121 | 10.69 | 1225 | 144 | 218, 145 | 1 |
| 58 | Xylitol | 5 TMS | 518901 | 17.24 | 1750 | 217 | 307, 205 | 1 |

^a^Level of metabolite identification according to Sumner et al. (2007). Levels being: (1) Identified compounds; (2) Putatively annotated compounds; (3) Putatively characterized compound classes; (4) Unknown compounds

^b^According GMD/NIST library

**Table S2.** Raw data of the performed experiment.

**Figure S1.** Silylation of arginine using MSTFA results in formation of ornithine 4TMS whereas the silylation product of agmatine is putrescine 4TMS. Both molecules lose a guanidine group during silylation. Hence the resulting sum parameters putrescine/agmatine and ornithine/arginine are reported in this study.

**Figure S2.** Altered metabolite levels 48 hours after deoxynivalenol treatment on the wheat cultivar ‘Remus’, which is highly susceptible towards Fusarium infection. Note that metabolites indicated with an asterisk* showed a significant difference with p<0.05 whereas ** identifies statistical significance even after multiple testing correction (Sidak; p <0.00091). For metabolites which were not detected (“n.d.”) in one of the two treatments no p-value could be calculated. The box represents the first and third quartile while the whiskers cover 99% of the data. Outliers are depicted as points.

**Figure S3.** Time courses of all 58 metabolites detected in this wheat experiment. DON treatment is depicted in black, whereas mock treated samples are blue; whiskers describe the standard deviation.
