# Supplementary figures and images for: GC–MS based targeted metabolic profiling identifies changes in the wheat metabolome following deoxynivalenol treatment
Source: Metabolomics. 2014 Sep 27;11(3):722–38. doi: 10.1007/s11306-014-0731-1 (PMC4419159; doi:10.1007/s11306-014-0731-1)

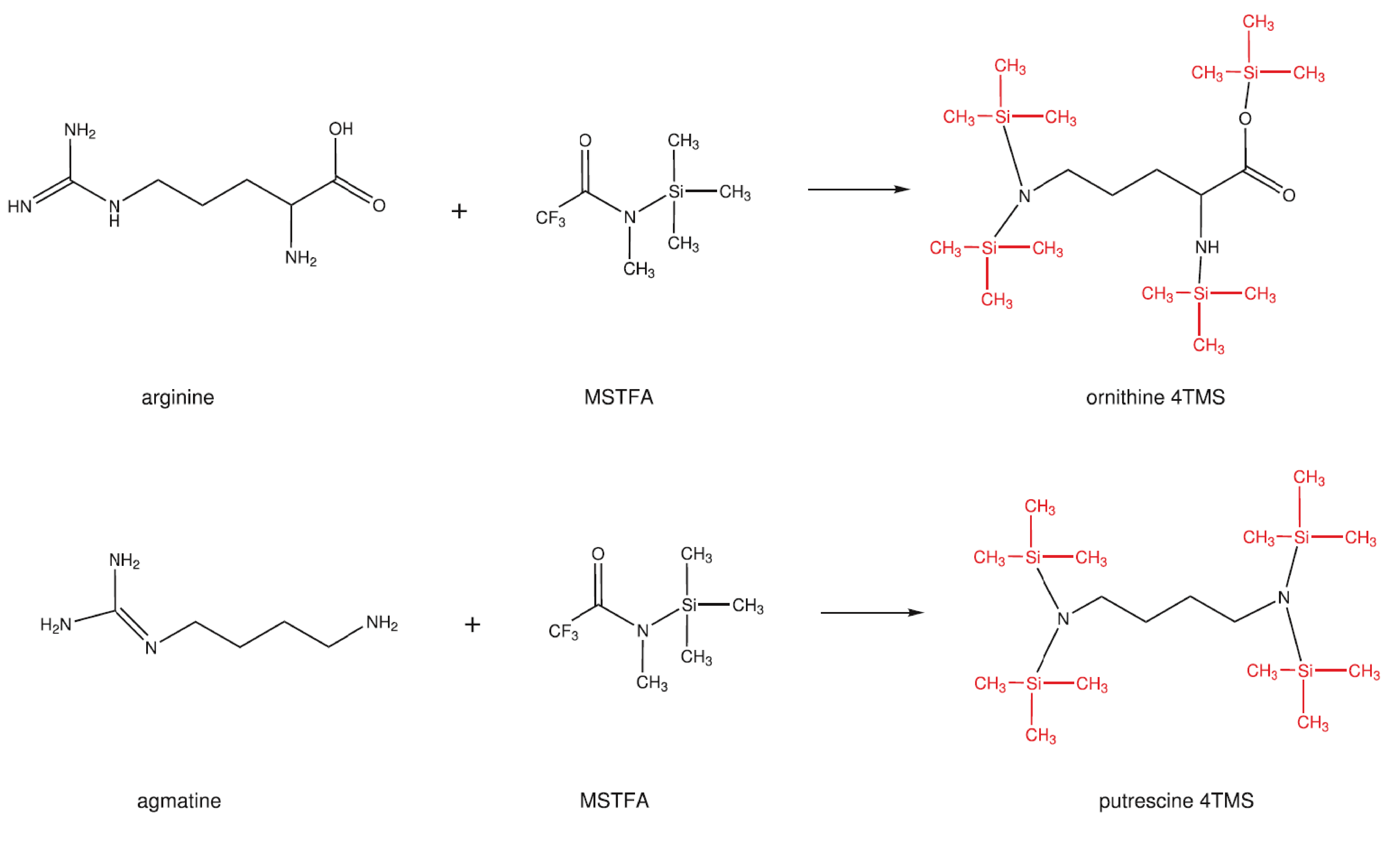

Supplement: Supplementary file 2 — Supplementary material 2 (TIFF 1396 kb) [file 11306_2014_731_MOESM2_ESM.tif]

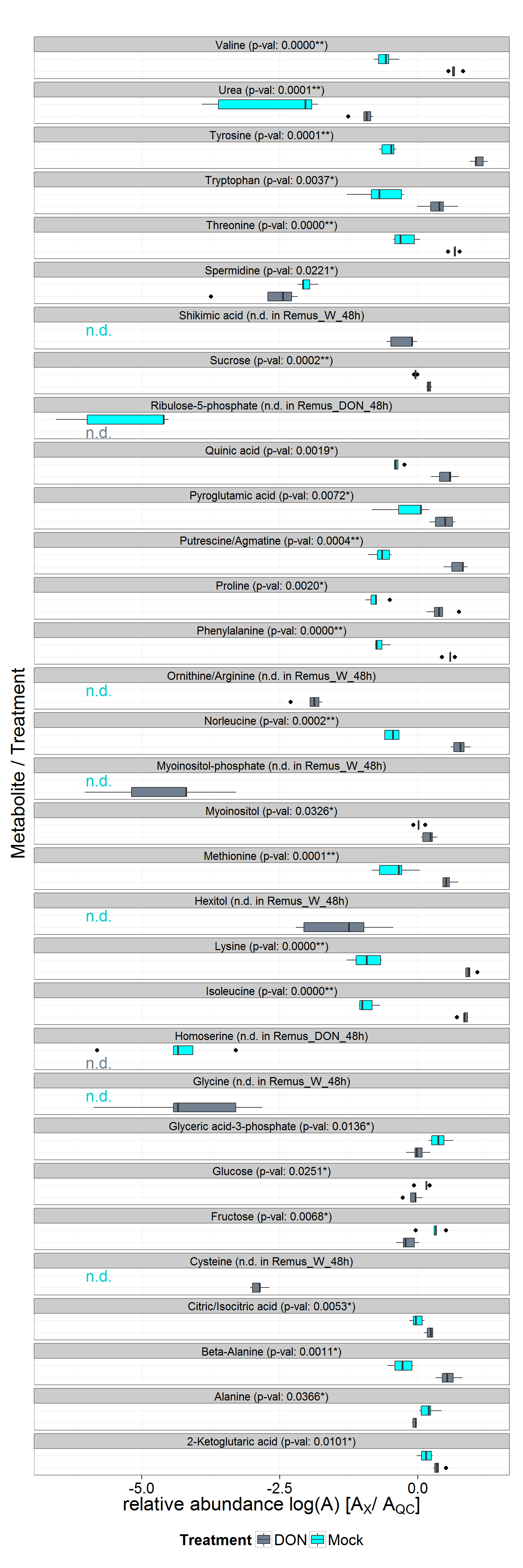

Supplement: Supplementary file 3 — Supplementary material 3 (TIFF 269 kb) [file 11306_2014_731_MOESM3_ESM.tiff]

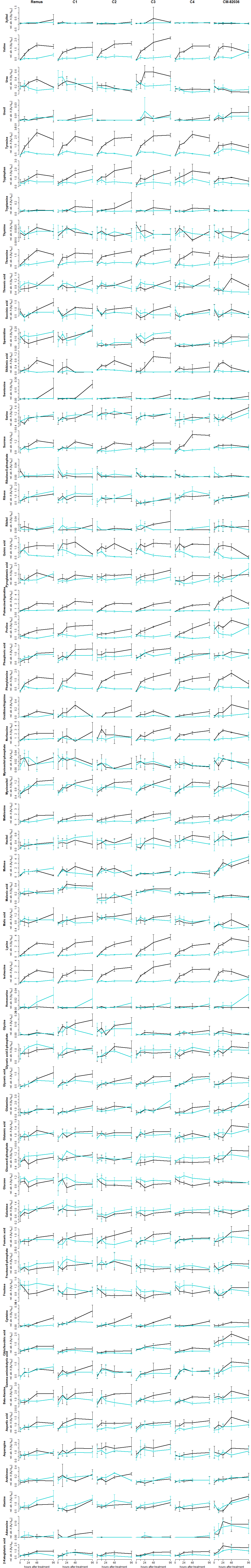

Supplement: Supplementary file 4 — Supplementary material 4 (TIFF 316 kb) [file 11306_2014_731_MOESM4_ESM.tiff]
